# Supplementary material for: Enhancing CO2 electrolysis through synergistic control of non-stoichiometry and doping to tune cathode surface structures
Source: Nat Commun. 2017 Mar 16;8:14785. doi: 10.1038/ncomms14785 (PMC5357311; doi:10.1038/ncomms14785)
Supplement: Supplementary Information — Supplementary Figures and Supplementary Tables [file ncomms14785-s1.pdf]

Supplementary Figure 1 XRD rietveld refinement patterns. (a) the oxidized LST_{O+}; (b) the reduced LST_{O+}; (c) the oxidized $LSTM_{O+}$; (d) the reduced $LSTM_{O+}$; (e) the oxidized $LSTC_{O+}$; (f) the reduced $LSTC_{O+}$.

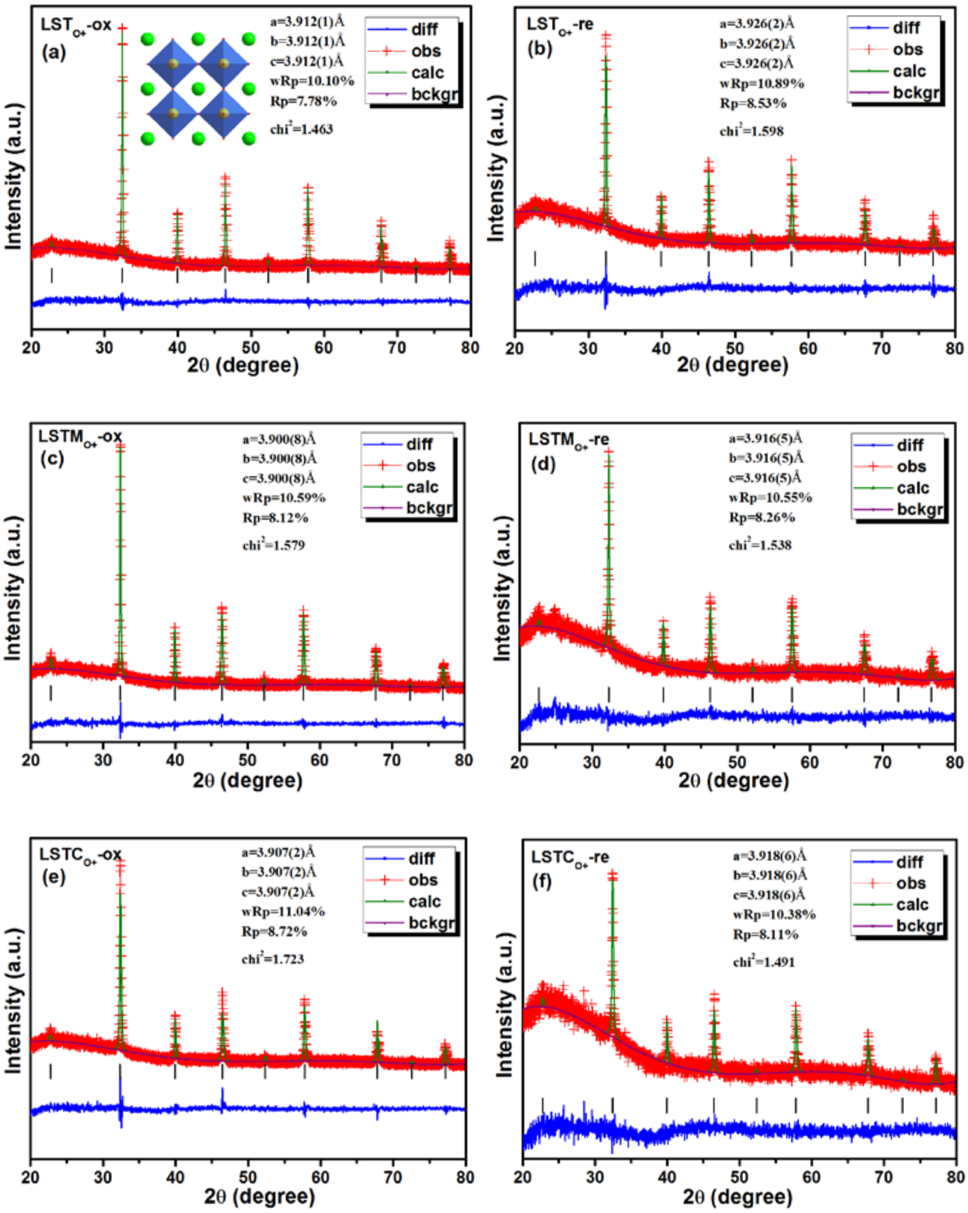

Supplementary Figure 2 XRD rietveld refinement patterns. (a) the oxidized $LSTMN_{A-}$; (b) the reduced $LSTMN_{A-}$; (c) the oxidized $LSTCN_{A-}$; (d) the reduced $LSTCN_{A-}$.

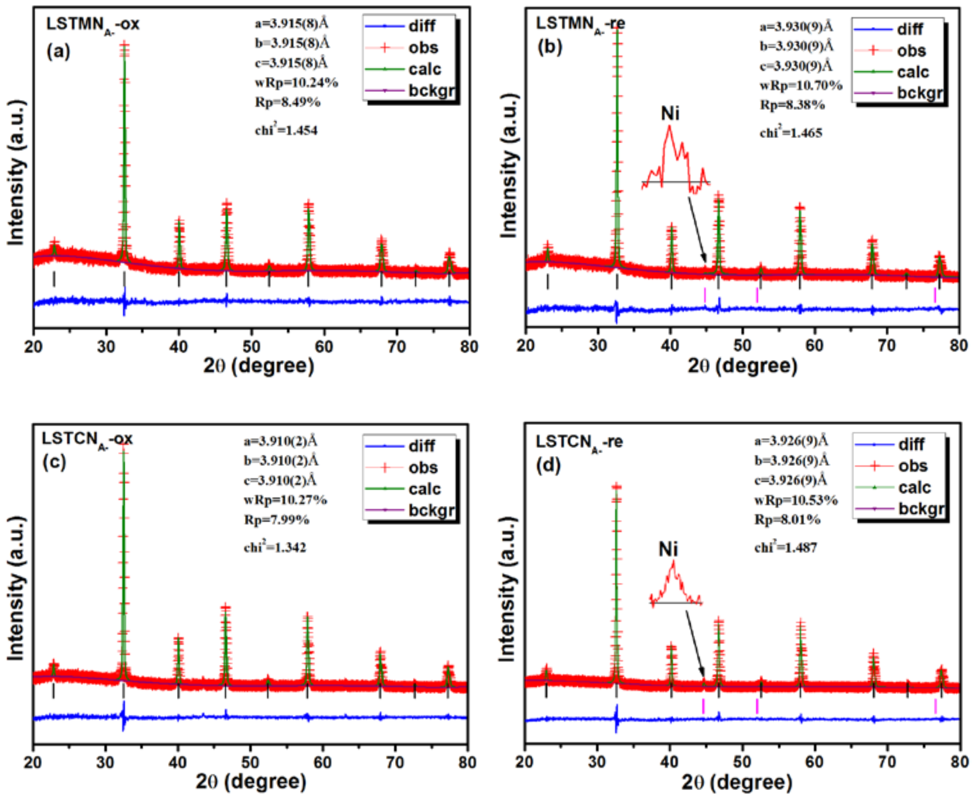

Supplementary Figure 3 X-ray photoelectron spectroscopy for $LSTMN_{A-}$ sample. XPS results of (a) Ti, (c) Mn, (e) Ni in the oxidized $LSTMN_{A-}$; (b) Ti, (d) Mn, (f) Ni in the reduced $LSTMN_{A-}$.

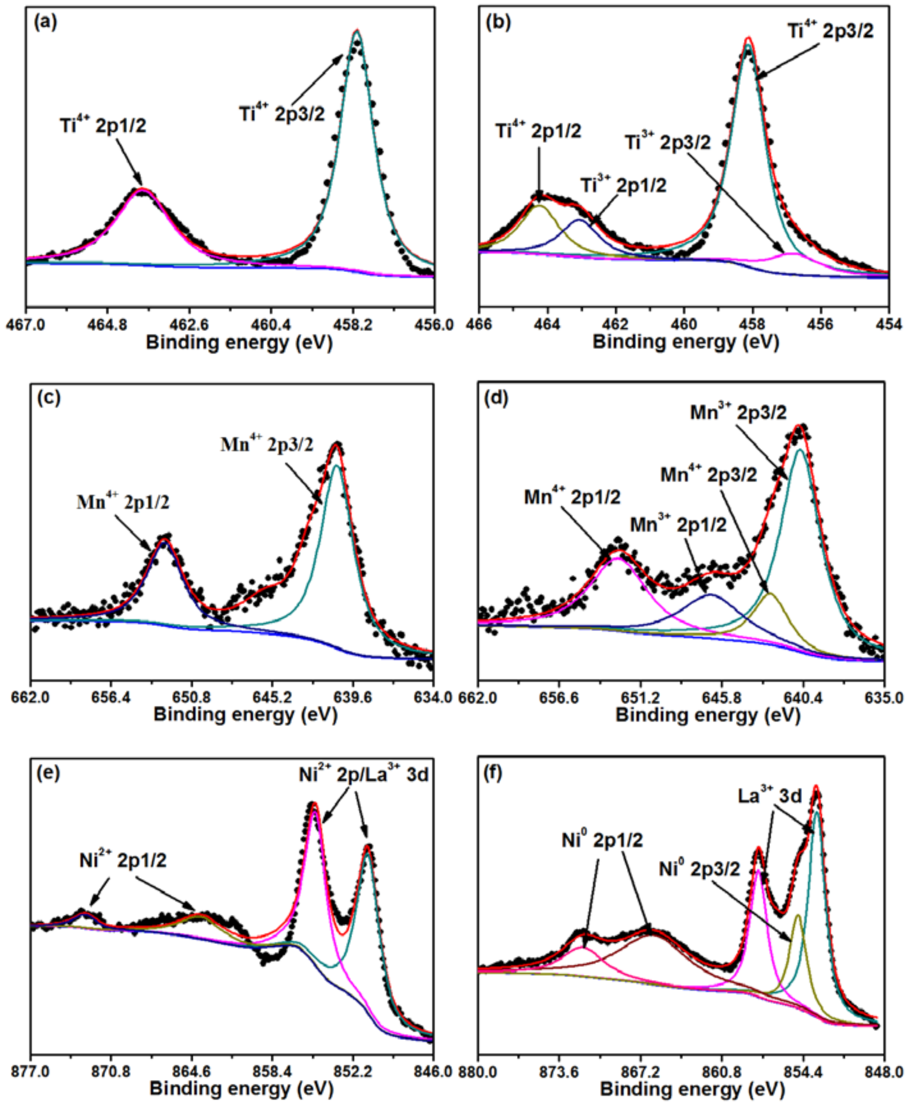

Supplementary Figure 4 X-ray photoelectron spectroscopy for $LSTCN_{A-}$ sample. XPS results of (a) Ti, (c) Cr, (e) Ni in the oxidized $LSTCN_{A-}$; (b) Ti, (d) Cr, (f) Ni in the reduced $LSTCN_{A-}$.

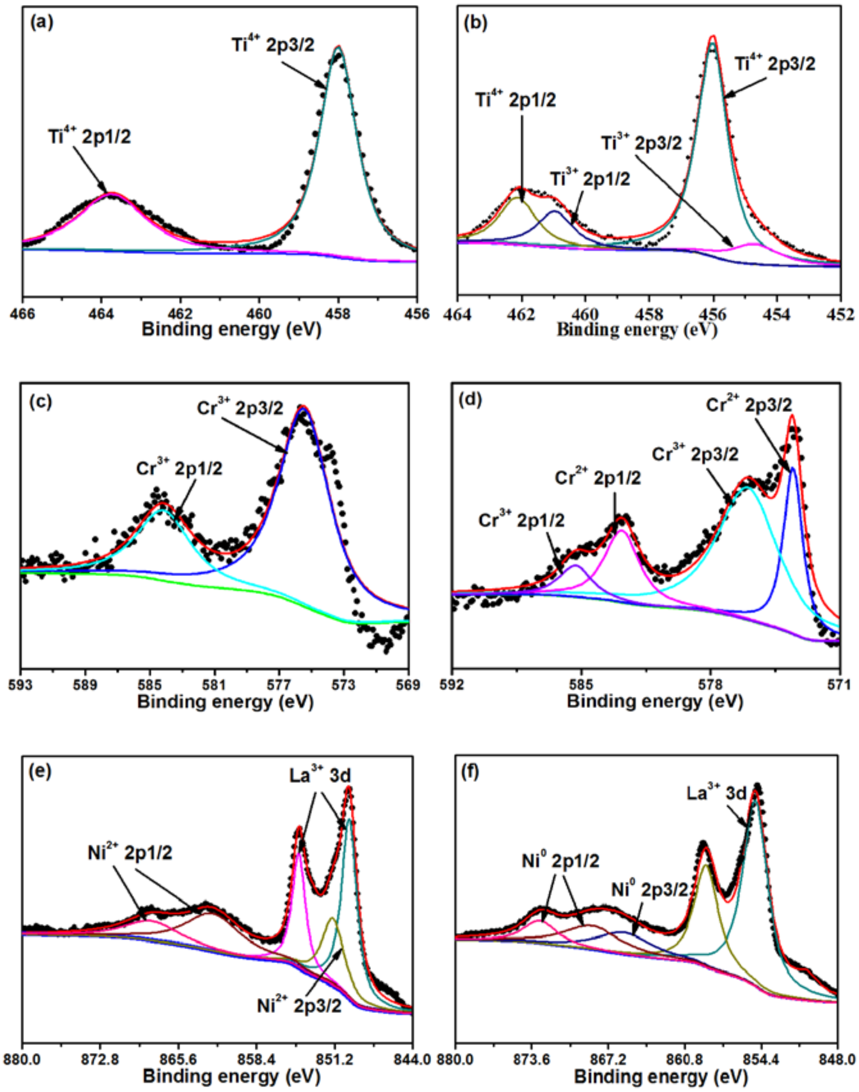

Supplementary Figure 5 Scanning Electron Microscopy. (a) The size distribution of 100 randomly selected Ni nanoparticles from the SEM results for the reduced $LSTMN_{A-}$; (b) SAED analysis for the reduced $LSTMN_{A-}$.

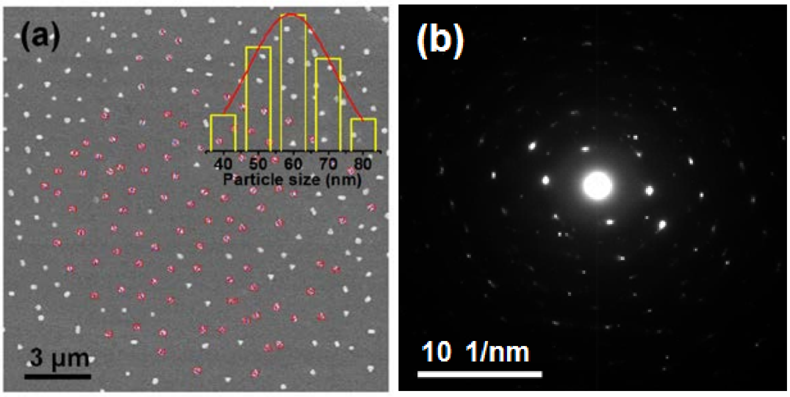

Supplementary Figure 6 Thermogravimetric analysis. (a) TGA tests of the reduced samples (800 °C for 20 h in 5%H_2/Ar) from 100 to 1200°C in air; (b) TGA tests of the reduced samples (strong reduction at 1200-1300 °C in 5%H_2/Ar) from 100 to 1200°C in air; (c and d) The raw data (b) of export diagrams.

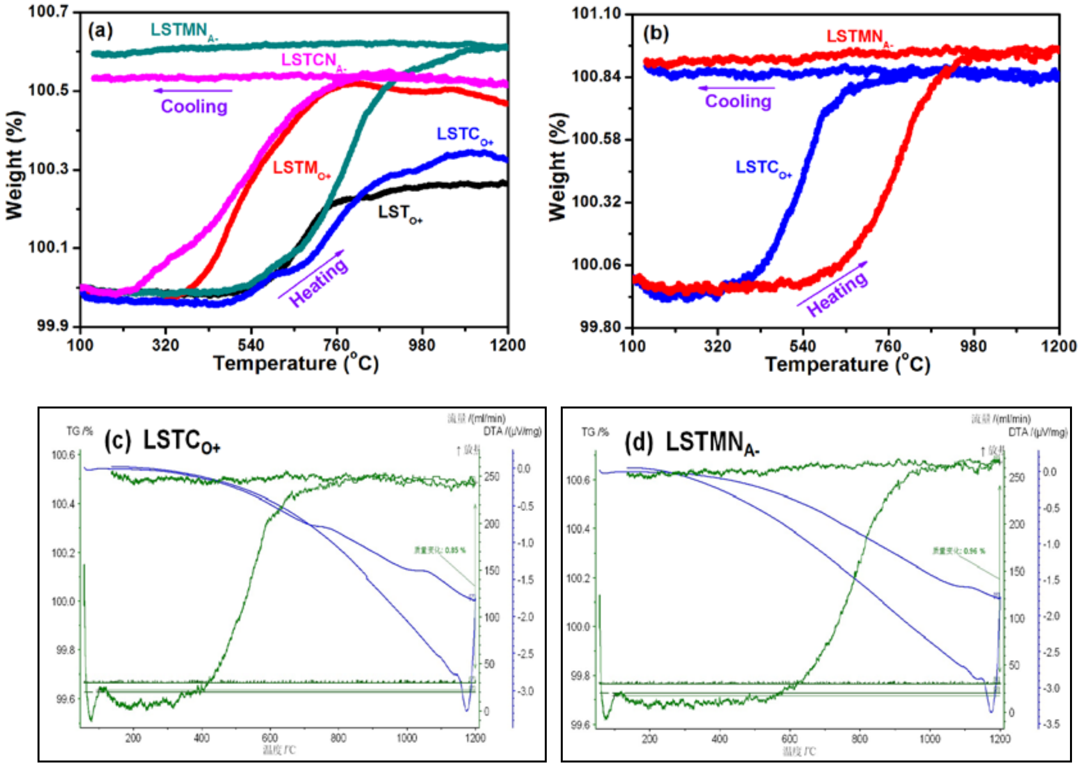

Supplementary Figure 7 Ionic conductivity and activation energy barrier of oxygen vacancy. (a) The dependence of ionic conductivity on temperature of the reduced samples in 5 % H_2/Ar; (b) the activation energy barrier of oxygen vacancy transfer process through two nearest oxygen sites.

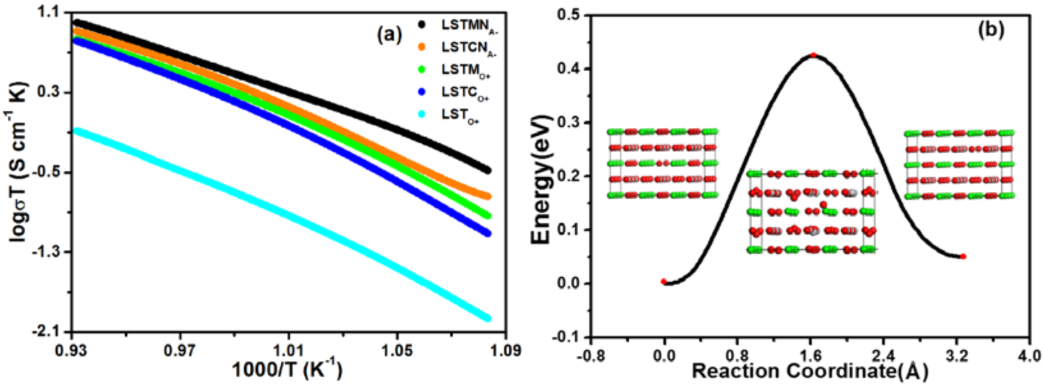

To investigate the possible oxide ion conduction mechanism in titanate, we simplified titanate as $SrTiO_3$ (STO) and calculated the activation energy barrier of oxygen ion transfer process in bulk. As oxygen may transfer through oxygen vacancies or interstitial sites in STO bulk, we consider both of these two situations. For facilitating the calculation, the oxygen vacancy transfer process is simplified as one oxygen vacancy transferring through two nearest oxygen sites as presented in Fig.S7 (b). As there is no traversable oxygen ion channel between two adjacent interstitial spaces in STO bulk, we studied a simplified interstitial oxygen transfer process, in which one interstitial oxygen atom moves to its adjacent oxygen site while the original oxygen atom in this site moves to another adjacent interstitial space simultaneously (Fig.S8). By employing the CI-NEB method, we calculated the energy barrier of these two oxygen transfer processes. As shown in Fig.S7 (b), the energy barrier of oxygen-vacancy transfer between the first and second adjacent oxygen atom sites is only 0.42 eV, which is much lower than the activation energy barrier for the interstitial oxygen transfer process (5eV, Fig.S8). As the Mn/Cr dopant would create a certain amount of oxygen vacancies in STO bulk, we propose that the oxygen vacancy transfer process would be energetically more favourable in the bulk. Strong charge transfer in bulk would be greatly encouraged by the low activation energy barrier of oxygen vacancy transfer.

Supplementary Figure 8 Activation energy barrier of interstitial oxygen. The activation energy barrier of interstitial oxygen transfer process.

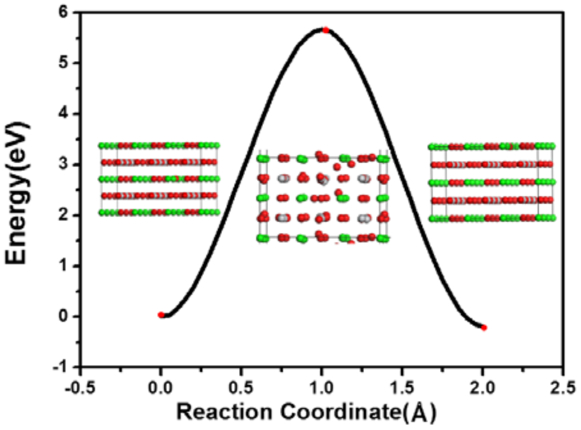

Supplementary Figure 9 Chemical adsorption of CO_2 for a series of samples. (a) and (b) *In situ* IR spectroscopy of the reduced samples without adsorbed CO_2 at room temperatures; (c) and (d) *In situ* IR spectroscopy of CO_2 adsorbed on the reduced samples at room temperatures; (e) TGA analysis of the reduced samples from 50 to 1000°C after CO_2 adsorption.

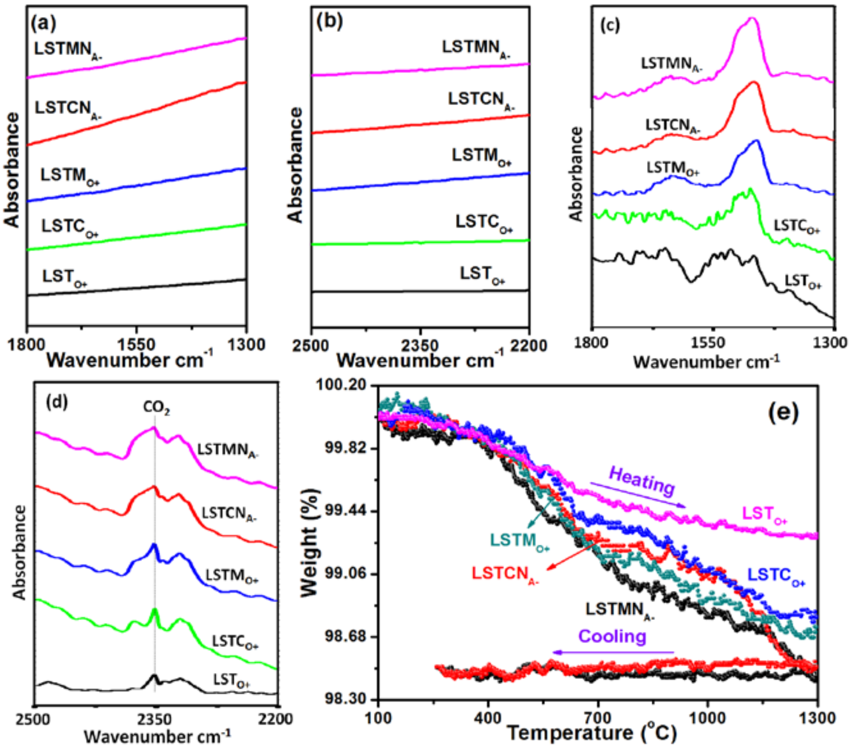

Supplementary Figure 10 The configurations of clean (110) Ni/STO system surface, and the left panels show top views while right panels give side views. Unit cells used in calculations are marked in blue rectangles. Nickel in blue Strontium is in green, titanium in pale, oxygen in red.

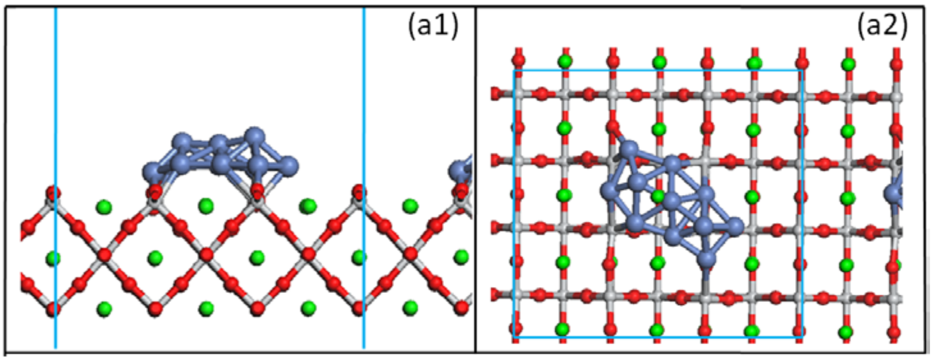

Supplementary Figure 11 Different chemisorption of CO_2 on the (110) Ni/STO system surface, and each CO_2 chemisorption energy is given on top of configuration with eV unit. Nickel in blue Strontium is in green, titanium in pale, oxygen in red, and carbon in gray.

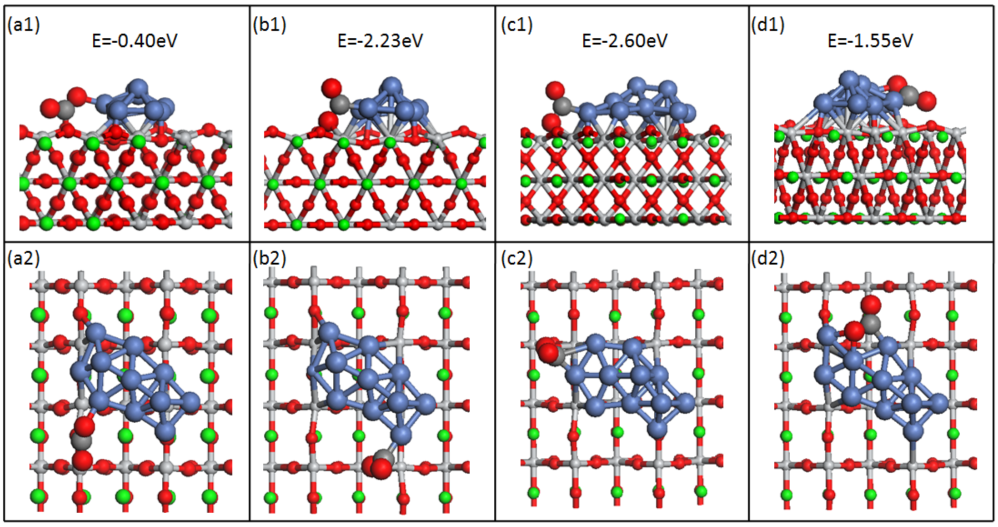

Supplementary Figure 12 (a) The original configurations of CO_2 approach to defected site of the (110) Ni/STO surface system; (b) the optimizational configurations of CO_2 approach to defected site of the (110) Ni/STO surface system. Left panels show top views while right panels give side views. Nickel in blue Strontium is in green, titanium in pale, oxygen in red, and carbon in gray.

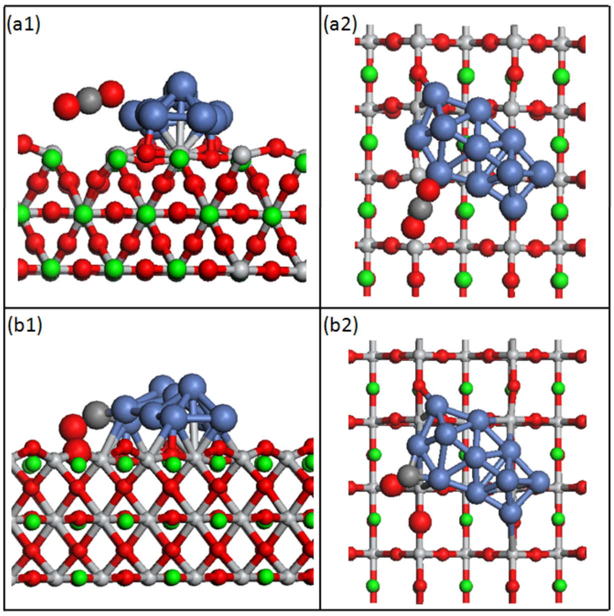

Supplementary Figure 13 Scanning Electron Microscopy for cells. Cross-sectional microstructure of the solid-oxide symmetric and electrolyzer cells: (a) LSTMN$_{A-}$/SDC-YSZ-LSTMN$_{A-}$/SDC; (b) LSTMN$_{A-}$/SDC-YSZ-LSM$_{A-}$/SDC.

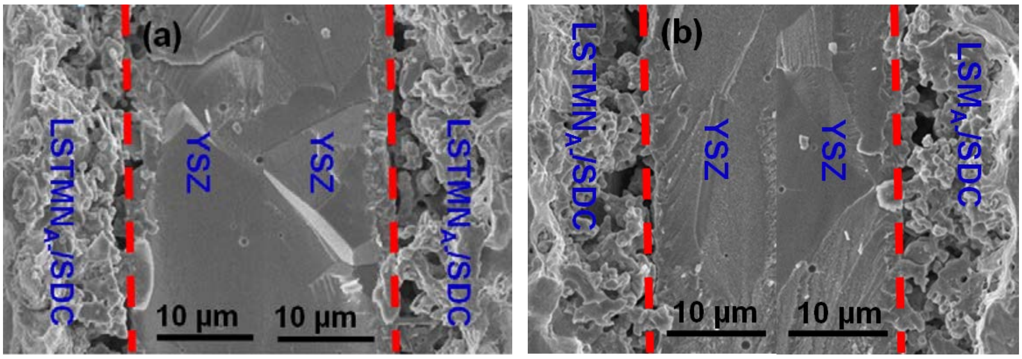

Supplementary Figure 14 AC impedance of the symmetric cells for a series samples. AC impedance of the symmetric cells for (a, b) LST_{O+}, (c, d) $LSTC_{O+}$, (e, f) $LSTM_{O+}$ at various H_2 partial pressures at 800°C.

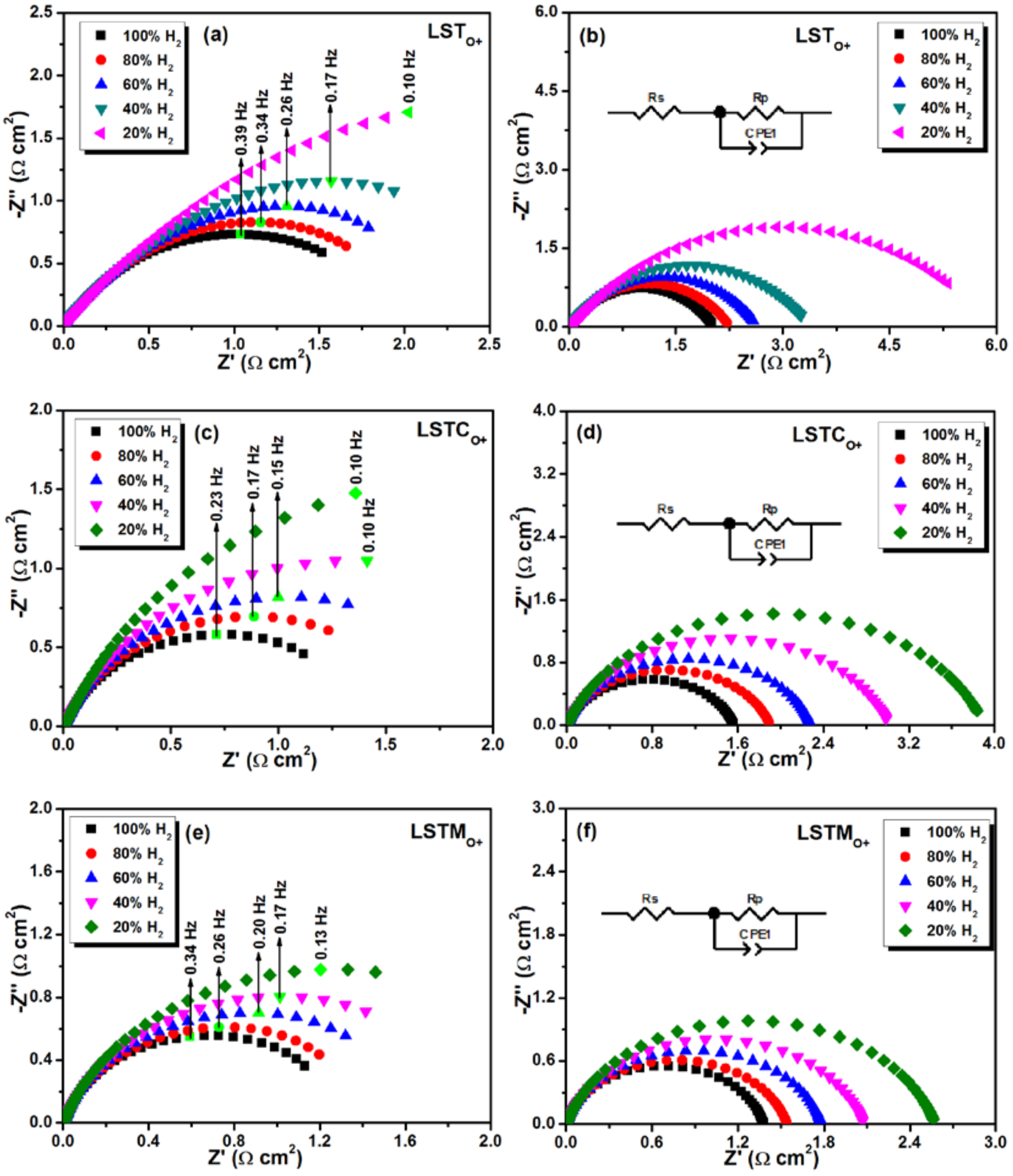

Supplementary Figure 15 AC impedance of the symmetric cells for a series samples. AC impedance of the symmetric cells for (a, b) $LSTCN_{A-}$ and (c, d) $LSTMN_{A-}$ at various H_2 partial pressures at 800°C.

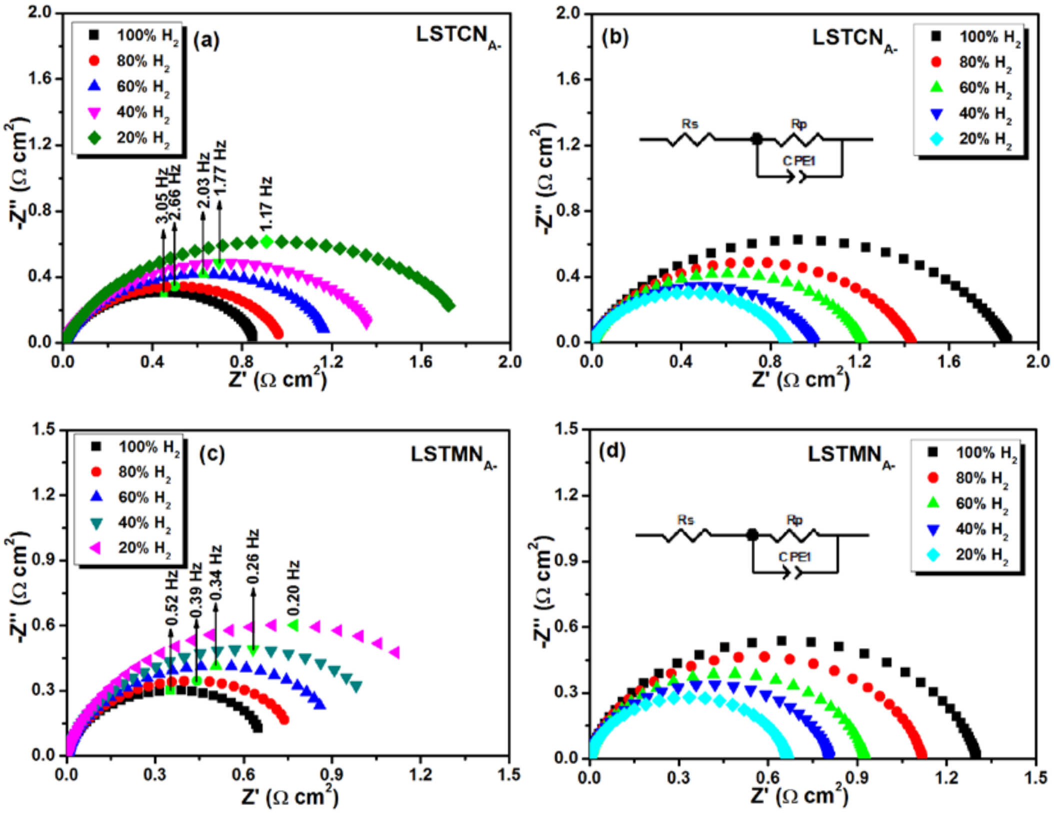

Supplementary Figure 16 AC impedance of the symmetric cells for a series samples. (a) The relationship between R_p with various H_2 partial pressures; (b) the AC impendence of the symmetric cells with different electrodes in 100 % of H_2 partial pressures at 800°C.

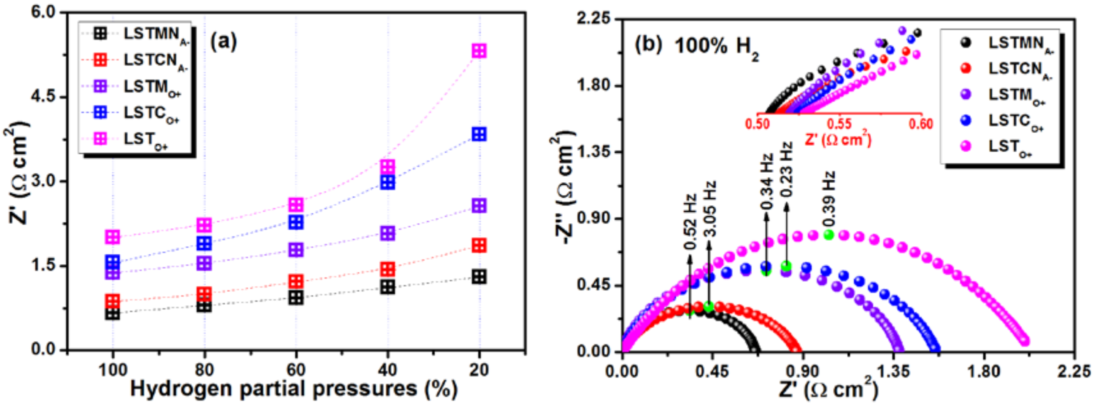

Supplementary Figure 17 Performance for 5% A-site deficiency. (a) XRD of oxidized and reduced samples; (b) and (c) *In situ* IR spectroscopy of CO_2 adsorbed on the reduced samples at room temperatures; (d) I-V curves of CO_2 electrolysis with different cathodes at 800°C; (e) short-term performances of CO_2 electrolysis at different voltages; (f) CO production and (g) current efficiency with different cathodes.

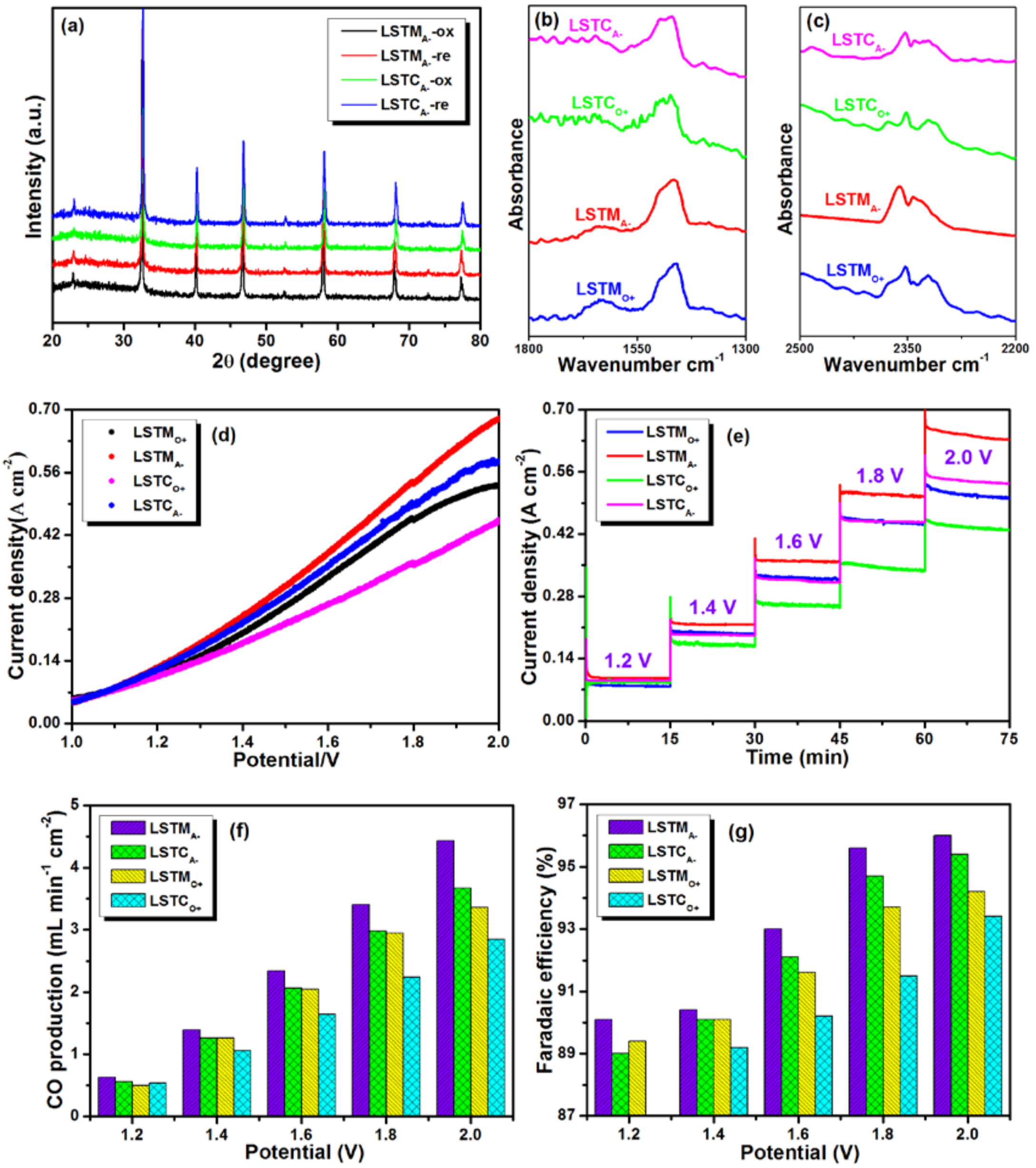

Supplementary Figure 18 *In situ* AC impedance spectroscopy of solid oxide electrolyser with different cathodes. AC impedance for electrolysers based on (a, b) LST_{O+}, (c, d) $LSTC_{O+}$, (e, f) $LSTM_{O+}$ at various voltages with the flow of CO_2 at 800°C.

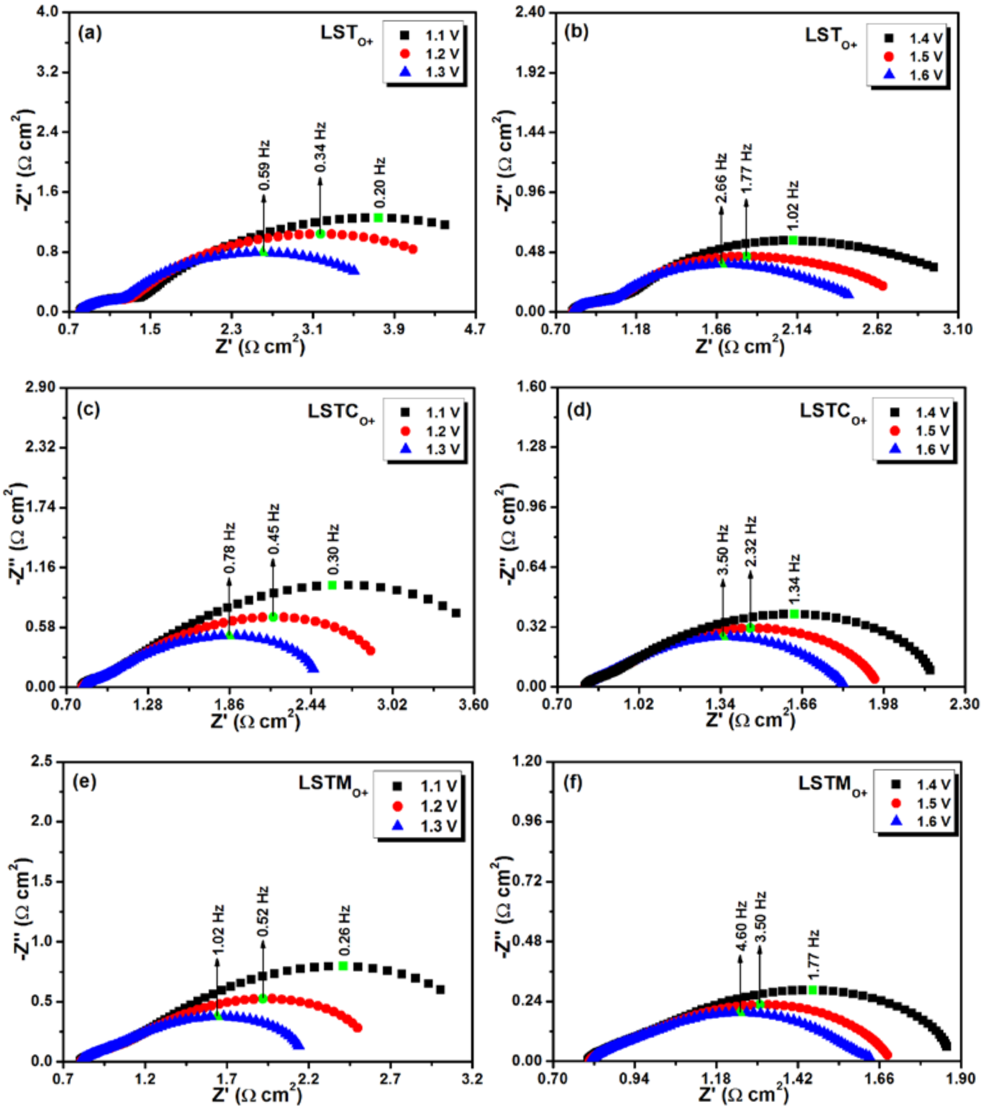

Supplementary Figure 19 *In situ* AC impedance spectroscopy of solid oxide electrolyser with different cathodes. *In situ* AC impendence for electrolyzers based on (a, b) $LSTMN_{A-}$ and (c, d) $LSTCN_{A-}$ at various voltages with the flow of CO_2 at 800°C.

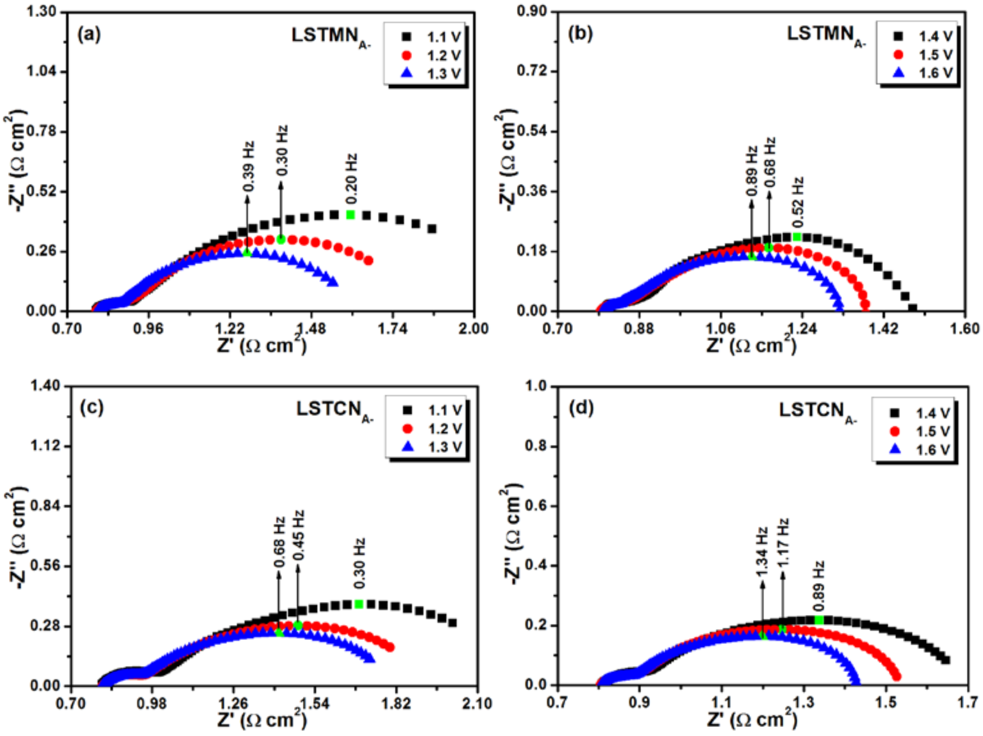

Supplementary Figure 20 Cycling performance for pure CO_2 electrolysis. The short-term performance of LSTMN$_{A-}$ cathode after 10 redox cycles.

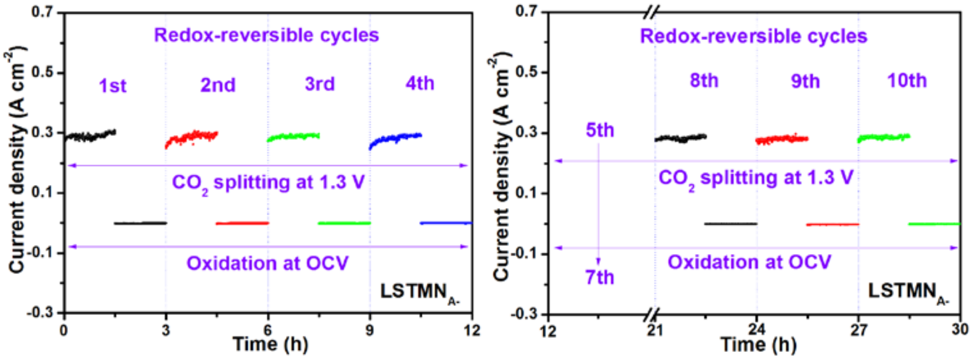

Supplementary Table 1 Cell parameters of the as-prepared samples.

Materials	Cell parameters (nm)	
	Oxidation	Reduction
LST_{O+}	3.912(1)	3.926(2)
$LSTM_{O+}$	3.900(8)	3.916(5)
$LSTC_{O+}$	3.907(2)	3.918(6)
$LSTMN_{A-}$	3.915(8)	3.930(9)
$LSTCN_{A-}$	3.910(2)	3.926(9)

Supplementary Table 2 Oxygen non-stoichiometry of different samples in oxidized or reduced states.

Materials	Oxygen content (mol)		Weight gain (%)
	Reduction	Oxidation	
LST_{O+}	3.068	3.100	0.26
$LSTM_{O+}$	3.044	3.100	0.46
$LSTC_{O+}$	3.011	3.050	0.32
$LSTMN_{A-}$	2.923	2.995	0.61
$LSTCN_{A-}$	2.885	2.945	0.51
